# A compact holographic projector module for high-resolution 3D multi-site two-photon photostimulation

**DOI:** 10.1371/journal.pone.0210564

**Published:** 2019-01-28

**Authors:** Mary Ann Go, Max Mueller, Michael Lawrence Castañares, Veronica Egger, Vincent R. Daria

**Affiliations:** 1 Department of Bioengineering, Imperial College London, South Kensington, SW7 2AZ London, United Kingdom; 2 Neurophysiology, Institute of Zoology, Universität Regensburg, 93040 Regensburg, Germany; 3 Eccles Institute of Neuroscience, John Curtin School of Medical Research, The Australian National University, Canberra, 0200 ACT, Australia; Stanford University, UNITED STATES

## Abstract

Patterned two-photon (2P) photolysis via holographic illumination is a powerful method to investigate neuronal function because of its capability to emulate multiple synaptic inputs in three dimensions (3D) simultaneously. However, like any optical system, holographic projectors have a finite space-bandwidth product that restricts the spatial range of patterned illumination or field-of-view (FOV) for a desired resolution. Such trade-off between holographic FOV and resolution restricts the coverage within a limited domain of the neuron’s dendritic tree to perform highly resolved patterned 2P photolysis on individual spines. Here, we integrate a holographic projector into a commercial 2P galvanometer-based 2D scanning microscope with an uncaging unit and extend the accessible holographic FOV by using the galvanometer scanning mirrors to reposition the holographic FOV arbitrarily across the imaging FOV. The projector system utilizes the microscope’s built-in imaging functions. Stimulation positions can be selected from within an acquired 3D image stack (the volume-of-interest, VOI) and the holographic projector then generates 3D illumination patterns with multiple uncaging foci. The imaging FOV of our system is 800×800 *μ*m^2^ within which a holographic VOI of 70×70×70 *μ*m^3^ can be chosen at arbitrary positions and also moved during experiments without moving the sample. We describe the design and alignment protocol as well as the custom software plugin that controls the 3D positioning of stimulation sites. We demonstrate the neurobiological application of the system by simultaneously uncaging glutamate at multiple spines within dendritic domains and consequently observing summation of postsynaptic potentials at the soma, eventually resulting in action potentials. At the same time, it is possible to perform two-photon Ca^2+^ imaging in 2D in the dendrite and thus to monitor synaptic Ca^2+^ entry in selected spines and also local regenerative events such as dendritic action potentials.

## Introduction

Two-photon (2P) microscopy is now widely used in neuroscience [1]. Its applications include structural and functional imaging [2–4], and neuronal stimulation by photolysis of caged chemical compounds and activation of light-gated ion channels [5, 6]. For example, the non-linear 2P excitation process allows highly localized photolysis of caged neurotransmitters, thereby enabling targeted activation of single spines and effectively mimicking synaptic inputs onto a neuron. By activating multiple synapses with this technique, we can study how neurons integrate synaptic inputs to generate action potentials. Because the dendritic arbor of a neuron extends beyond a single plane, it is highly desirable to have three-dimensional (3D) access to individual synaptic sites to provide experimental flexibility. For example, 3D access to a sufficiently large volume with high spatial resolution will allow to investigate synaptic integration across dendritic bifurcations for any spatial arrangement of the dendrites of interest.

Conventional laser scanning systems use galvanometer mirrors [7] that deliver efficient beam steering with minimal power loss across a wide field-of-view (FOV) of the sample. The mirrors can be programmed to perform a raster scan or to follow an arbitrary path [8] and have a positioning time of ∼ 100 *μ*s. Resonant scanning systems [9], which use a resonant mirror in one axis (fast axis) and a galvanometer mirror in the other axis, allow faster scan rates but are constrained to raster scans. Even faster beam steering (< 15 *μ*s [10]) is offered by two acousto-optic deflectors (AODs), which steer the laser beam without any mechanically moving parts. In these scanning systems, fast switching times from one stimulation site to another allow the near-simultaneous activation of multiple synapses in a physiologically relevant time window (∼ 1 ms). While these scanning systems are constrained to two dimensions (2D), high-speed 3D scanning using a system of four AODs has been demonstrated [11, 12]. However, this system is characterized by a low optical throughput (∼ 18%) [13]. Although such a system is useful for high-speed monitoring of neuronal activity by imaging fluorescent reporters (e.g. calcium indicators), so far it has not been applied to 2P uncaging because of this limitation in optical throughput.

Simultaneous steering of multiple laser beams via holographic projection offers a solution [14–22]. High optical throughput is afforded by using a phase-only spatial light modulator (SLM) to modulate the wavefront of the laser beam to produce a target intensity distribution at the objective’s focal plane. Phase holograms can produce spatial light patterns such as multiple diffraction-limited focal spots that are ideally suited for highly localized uncaging in synaptic integration studies. Such multi-focal patterns were initially used to demonstrate multi-site 2P glutamate uncaging in 2D [14–16]. Holographic projectors are inherently capable of generating multiple foci in 3D especially with computer-programmable SLMs. This has been shown with single-photon (1P) photolysis [17–20]. We have also previously demonstrated holographic projection to perform multi-site 2P uncaging [21, 22], which enabled us to stimulate multiple spines along dendrites in 3D with high spatial (transverse and axial) resolution. However, holographic projectors have finite spatial range or FOV for a desired resolution as quantified by the optical system’s space-bandwidth product. To ensure effective 2P uncaging, we needed highly resolved focal spots and the trade-off between FOV and resolution resulted in a limited spatial coverage. Such trade-off does not necessarily apply to 1P uncaging since there is sufficient light energy (e.g. ultra-violet light) to photolyse caged molecules without requiring tight focusing. However, expanding the holographic FOV with 1P uncaging also means a less resolved uncaging system that does not necessarily target individual spines. While such a 1P uncaging system may be applicable for some applications, specific neurobiological questions that involve integration of synaptic inputs from individual spines require a highly resolved multi-site 2P uncaging system.

Here, we extend the spatial coverage of a multi-site 2P uncaging system by integrating a compact holographic module into a commercial 2P laser scanning microscope. While our solution does not increase the holographic FOV, it enables us to use the microscope’s scanners to reposition the holographic FOV across a wider spatial range whilst maintaining highly resolved focal spots. We integrated the hardware and software components of the module with that of the microscope’s built-in functions (such as 3D rendering, arbitrary region of interest (ROI), zoom and panning functions) to perform synaptic integration experiments using the 3D image of the neuron’s dendritic tree. From the 3D image of the dendrites, we can calculate an appropriate phase-only hologram to accurately produce multi-focal stimulation sites along any dendritic arborization. We demonstrate the functionality of the system with 2P glutamate uncaging experiments at dendrites of cortical pyramidal neurons, targeting multiple spines in volumes of up to 70×70×16 *μ*m^3^ as dictated by the 3D morphology of the neurons we investigated (possible *z*-range up to 70 *μ*m). Using the galvanometer mirrors, we can reposition the holographic FOV within an 800×800 *μ*m^2^ imaging FOV. We can stimulate a sufficient number of spines to evoke an action potential from the neurons’ resting membrane potentials.

## Materials and methods

Our setup consists of a holographic projector integrated into a commercial 2P galvanometer-based scanning system with an uncaging unit (Femto2D-Uncage, Femtonics Ltd., Budapest). The system is equipped with two femtosecond pulsed Ti:S lasers (Chameleon Ultra I and II, Coherent) and is capable of simultaneous uncaging and calcium (Ca^2+^) imaging at different wavelengths. We describe the design and alignment of the holographic projector, the integration of its software control into the commercial software package, and its application to 2P uncaging experiments *in vitro*.

### Design of holographic projector attachment

**Fig 1:**
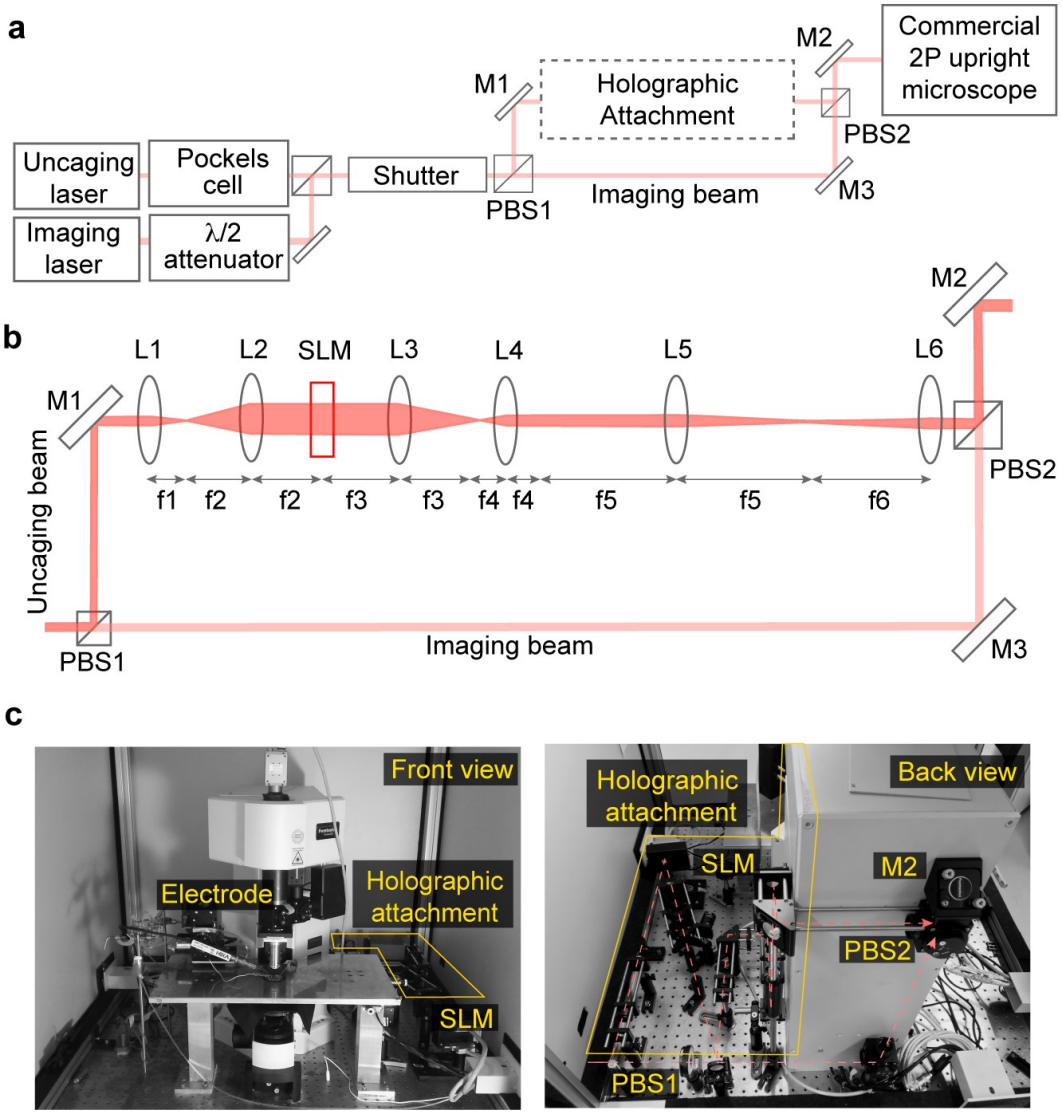
Optical setup of holographic projector attachment. **a**: Schematic of the commercial two-photon microscope and the holographic attachment. **b**: Detailed schematic of the holographic projector attachment. PBS: polarizing beam splitter, M: mirror, L: lens, SLM: spatial light modulator, λ/2: half waveplate. H1: conjugate image of the SLM hologram (H0). Lenses: *f*1 = *f*4 = 30 mm; *f*2 = *f*3 = 100 mm; *f*5 = *f*6 = 500 mm. **c**: Photographs of the setup showing the front view of the commercial microscope (left), and the back view showing the holographic attachment (right).

The schematic of the system and the design of the holographic projector are shown in Fig 1. We inserted a polarizing beam splitter (PBS1) after the intensity attenuation optics and safety shutters, just before the entrance port of the 2P upright microscope Fig 1a. Because the polarizations of the two lasers are perpendicular with respect to each other, PBS1 relays the uncaging beam to the holographic projector while transmitting the imaging beam. The uncaging beam is expanded by a 4*f* lens setup (L1, L2) to illuminate the 15.8×12 mm^2^ window of a reflective phase-only SLM (X10468-03, Hamamatsu, Japan) at an angle of ∼ 18° from the normal Fig 1b. The SLM has a resolution of 800×600 pixels, but only the central 600×600 pixels are used to display the phase-only hologram (H0). A demagnifying 4*f* lens setup (L3, L4) reduces the uncaging beam width back to its original size setting the conjugate image of the SLM or hologram plane at the focus of L4 (H1). To efficiently couple this phase-encoded beam to the microscope, another appropriate 4*f* relay optics (L5, L6) has to be chosen such that the conjugate plane of the hologram (H1) is imaged on to the plane of the galvanometer scanning mirrors (GM) of the microscope. This setup ensures that all beams deflected by the phase hologram are contained within the area of the scanning mirrors. The distance from the GM unit to the entrance port of the commercial microscope is ∼ 500 mm, determining the focal length of the 4*f* relay lenses L5 and L6. After lens L6, we placed a second polarizing beam splitter (PBS2) to recombine both the imaging and uncaging beams. Both beams are relayed onto the GM through the scan and tube lenses, and finally the objective lens (20×, NA 1.0, W Plan-Apo, Zeiss or 60×, NA 1.0 W, NIR Apo, Nikon). The scan lens and the tube lens of the microscope likewise image the hologram onto the back aperture of the objective lens. With the exception of L3 (diameter ⊘ = 50.8 mm), all optical components have a diameter of 25.4 mm and fit into a 30 mm cage system. As evident from Fig 1c the holographic projector design is rather compact, requiring no more than 80×35 cm^2^ base area.

### Software control of holographic attachment

**Fig 2:**
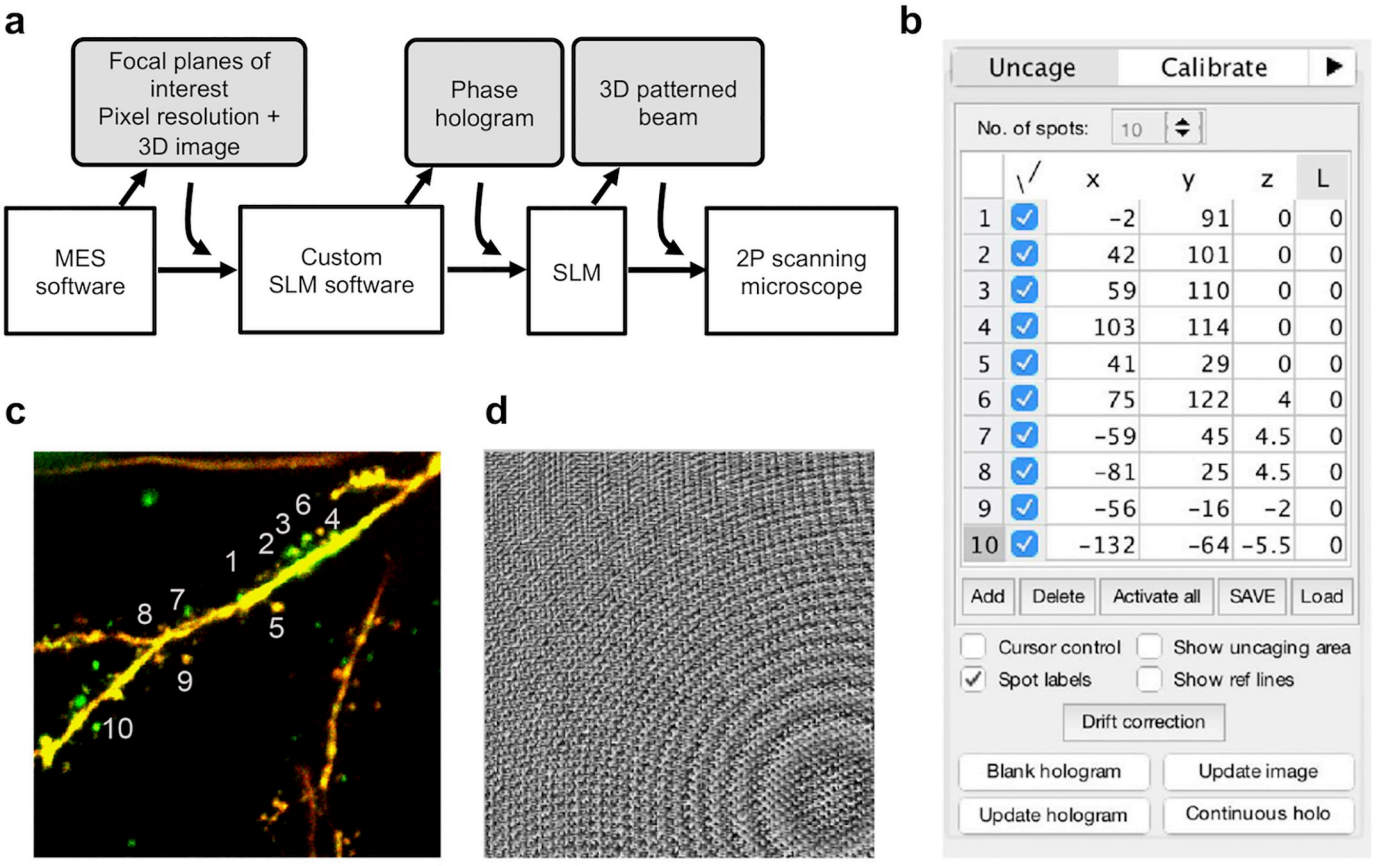
Software control of holographic projector. **a**: Interfacing between our custom SLM software and the rest of the system including the microscope’s control software (MES), which gathers the pixel resolution. **b**: Custom software graphical user interface showing *xyz*-coordinates of uncaging spots. We use the last column to encode a Laguerre-Gaussian beam of charge “L” to align the center of the SLM with respect to the optical axis. **c**: Two-photon 3D image of a basal dendrite with multiple spines. Positions of uncaging sites in **b** are indicated. **d**: The corresponding 8-bit phase-only hologram for projecting the multiple uncaging spots in **b**.

In our case, the commercial microscope comes with a MATLAB-based software package, MES (Femtonics Ltd.), which controls the microscope and acquires 3D images within the volume-of-interest (VOI) set by the user. We developed a custom software plugin written in MATLAB (v2007b, Mathworks) to interface with the commercial software and to generate the corresponding hologram for the arbitrary positioning of multiple foci for uncaging. Fig 2a shows how the custom software interfaces with the commercial software and the rest of the system.

For each focal plane in the VOI, the user first takes an image via the MES software, which stores the image and its metadata in the hidden global MATLAB workspace. The custom SLM software reads the acquired image and its pixel resolution. The user then positions the uncaging sites by specifying the *xy* coordinates on the graphical user interface (GUI, Fig 2b) or by positioning crosshairs on the image (Fig 2c). Next, the user adjusts the *z*-coordinates of the spots depending on their relative distance to the plane where the objective will be focused during photostimulation. These *xyz* coordinates are then used to calculate a hologram, based on the standard prism-lens superposition algorithm [23, 24], which is displayed on the SLM (Fig 2d) via a digital video interface (DVI) using the PsychoPhysics Toolbox in Matlab. The phase hologram on the SLM is the phase, arg {*ψ*(*u*, *v*)}, of the total input field, *ψ*(*u*, *v*), described by

$$\begin{array}{c} \psi \left(u,v\right)=\sum_{n=1}^{N}A_{n}exp\left(2\pi i\left[\alpha ux_{n}+\beta vy_{n}+\gamma \left(x^{2}+y^{2}\right)z_{n}+\sum_{p,q}C_{q}^{p}\left(z_{n}\right)Z_{q}^{p}\left(u,v\right)\right]\right), \end{array}$$ (1)

where *A*_*n*_ and (*x*_*n*_, *y*_*n*_, *z*_*n*_) are the intensity weighting coefficient and 3D spatial coordinates, respectively, of the *n*th uncaging spot, (*u*, *v*) are Cartesian coordinates at the SLM plane with the center at the optical axis and *α*, *β*, *γ* are scaling factors which take into account the excitation wavelength, objective focal length and calibration adjustments [21, 25]. $C_{q}^{p}\left(z_{n}\right)$ and $Z_{q}^{p}\left(u,v\right)$ are the Zernike coefficients and polynomials, respectively, used to compensate for optical aberrations.

The SLM-generated excitation spots may be degraded due to optical aberrations introduced along the optical path. These aberrations can be mathematically represented by Zernike polynomials [26], which can be encoded onto the SLM in addition to the calculated phase hologram. To correct for aberrations, the user simply has to adjust the coefficients for the Zernike polynomials in the custom software GUI (S1a Fig). The user may also correct for drift in the 2P image by adjusting the positions of the uncaging spots on the GUI.

The MATLAB code for the software plugin and details on how to use it in conjunction with MES are available at http://www.github.com/g0codes/holoMES.

### Alignment and calibration of excitation spots

We used the imaging beam as a reference and aligned the uncaging beam to it via two alignment irises. For fine adjustment, we imaged 1 *μ*m diameter fluorescent beads (Molecular Probes, Inc.) with the 60× objective using both lasers at 750 nm simultaneously but with a blank hologram displayed on the SLM. This double illumination produces two images of the beads if the uncaging beam is not completely aligned to the imaging beam. Fine adjustments in alignment were made by adjusting the direction of the uncaging beam via relay optics just before PBS2 in Fig 1a).

To calibrate the position of the holographic excitation spots, we used mirror images of a bead resulting from scanning a patterned uncaging beam onto the bead sample (without the imaging beam). For instance, imaging a single fluorescent bead with a two-foci uncaging beam results in two mirror images. We imaged fluorescent beads this way while adjusting the *x*, *y* and *z* scaling and rotation factors of the hologram until the mirror images of the beads on the 2P image were centered on the uncaging cross hairs on the GUI (S1b Fig).

We measured the 2P point spread function (PSF) of our system by imaging 100 nm fluorescent beads (Molecular Probes, Inc.) with the 60× and 20× objective lenses, using both imaging and uncaging lasers separately.

### Acute brain slice preparation and electrophysiology

All experimental procedures were approved by the University of Regensburg Ethics Committee (Egger Ethics ID: AZ 55.2-1-54-2532.2-58-11). Sagittal somatosensory cortex acute brain slices (300 *μ*m thick) from juvenile rats (postnatal days 11-18, Wistar) were incubated at 33°C for 30 min in ACSF bubbled with carbogen and containing (in mM): 125 NaCl, 26 NaHCO_2_, 1.25 NaH_2_PO_4_, 20 glucose, 2.5 KCl, 1 MgCl_2_ and 2 CaCl_2_. Recordings were performed at room temperature (22°C). Patch pipettes (pipette resistance 4-5 MΩ) were filled with an intracellular solution containing (in mM): 140 K gluconate, 10 HEPES, 10 NaCl, 0.5 MgCl_2_, 4 Mg-ATP, 0.4 Na_3_-GTP, 0.1 OGB-1 and 0.04-0.06 Alexa Fluor 594, pH 7.3.

Electrophysiological recordings were made using an EPC-10 amplifier with Patchmaster v2.60 software (HEKA). Layer 2/3 pyramidal neurons were patched in whole-cell configuration and held in current-clamp mode close to a membrane potential of -60 to -75 mV. Electrophysiological data were analyzed using Igor Pro (Wavemetrics). Somatic recordings of both single spine stimulations and the different combinations of multi-site uncaging were averaged for each stimulation type (*n* = 2).

### Two-photon imaging and glutamate uncaging

To visualize a neuron, we loaded 100 *μ*M OGB-1 (Ca^2+^ indicator, Life Technologies) and 40-60 *μ*M Alexa Fluor 594 (Life Technologies) into the patch pipette and patched the cell in whole-cell configuration. After waiting for the dye to diffuse into the dendrites (∼ 20 mins), a 2P image stack of the neuron was recorded by the commercial software (MES, Femtonics) with the imaging laser set to an excitation wavelength of 835 nm. We loaded 1 mM DNI-caged glutamate (DNI, Femtonics Ltd.) via a closed perfusion circuit with a total volume of 12 ml ACSF. The solution containing the caged glutamate was washed in for 10 min prior to photostimulation. The uncaging wavelength was 750 nm while exposure time was 0.5-1.5 ms and laser power was adjusted individually for each experiment to elicit physiological responses. Using the custom-written SLM software, uncaging spots were positioned in 3D at a distance of ∼ 0.5 *μ*m from spine heads. *z*-positions of the uncaging spots were first estimated from the 3D image, then further adjusted to obtain an optimal excitatory postsynaptic potential (EPSP) response. Positions were checked before each measurement and, if necessary, readjusted to account for drift. Imaging of uncaging-evoked Ca^2+^ signals in selected spines was carried out as described earlier [27]. During simultaneous Ca^2+^ imaging and photostimulation, imaging was started 700 ms before the uncaging stimulus during which the scanning mirrors were fixed. For simultaneous multi-site photostimulation in synaptic integration experiments, the total uncaging power and the number of foci were kept constant. “Superfluous” foci, i.e. foci that were not needed as stimulation spots at a given time of an experiment, were excluded by positioning them just outside the holographic FOV, such that they would fall off the optics and not be projected onto the sample.

## Results

**Fig 3:**
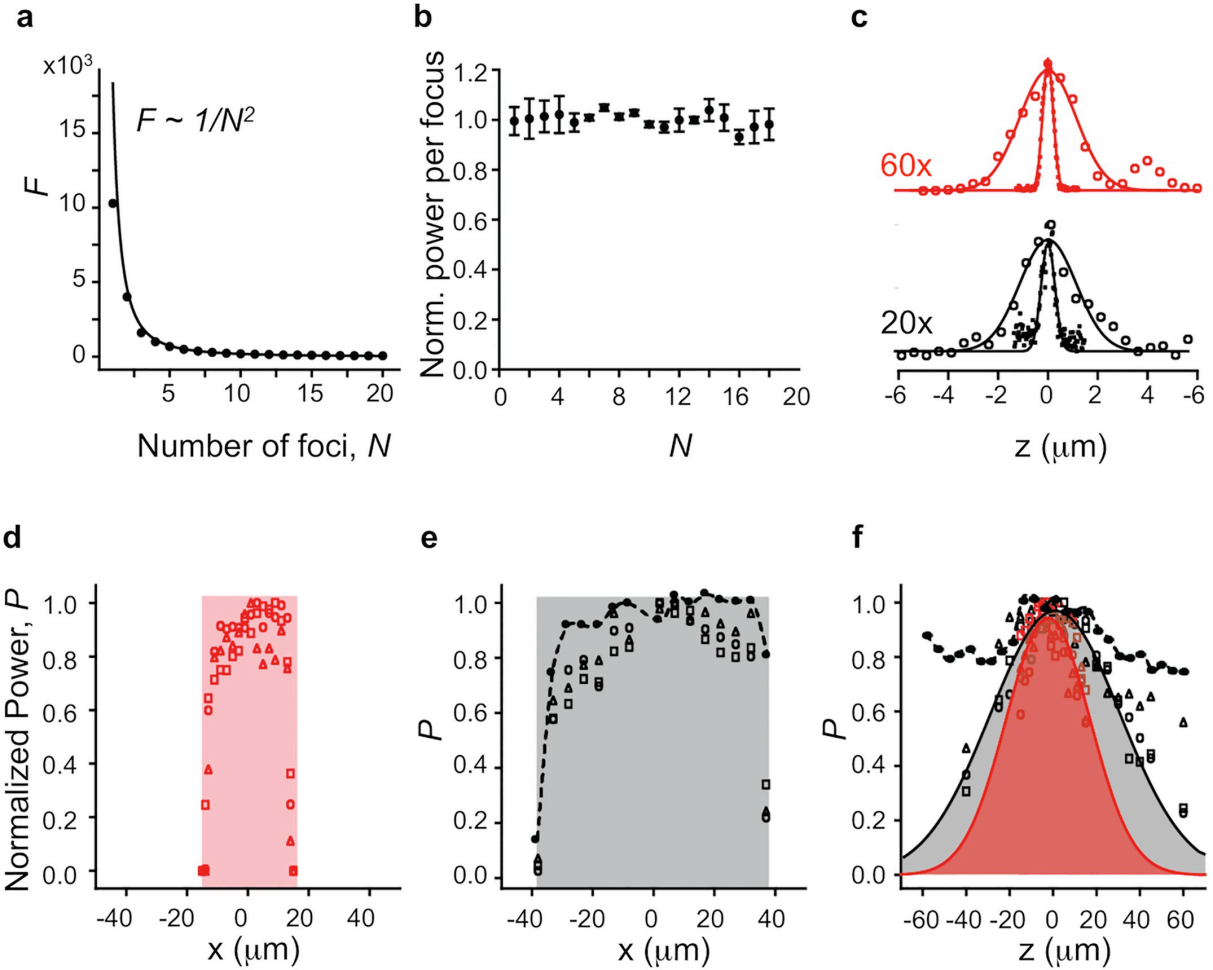
Performance of the holographic system. **a**: Normalized fluorescence intensity of a focal spot as a function of number of spots, *N* **b**: Normalized uncaging power per spot as a function of number of stimulation spots. Number of foci is kept constant and superfluous foci are moved outside of the holographic FOV in transverse direction. **c**: Measured PSF of two-photon excitation in the axial (⚬) and transverse (●) directions for 60× and 20× objective lenses. **d**: Normalized power as a function of displacement along the *x* for 60× for FOV centered at (0, 0) (⚬), (−100, −100) *μ*m (△) and (100, 100) *μ*m (□). **e**: Normalized power as a function of displacement along the *x* for 20× for FOV centered at (0, 0) (⚬), (−300, −300) *μ*m (△) and (300, 300) *μ*m (□). Filled circles with dashed line denote power distribution after amplitude weighting for FOV centered at (0, 0). **f**: Normalized power as a function of displacement along the *z* for 60× (red markers in red area) and 20× (black markers in gray area) for FOV centered at different coordinates specified in **d** and **e**. Solid lines denote empirical Gaussian fit used for amplitude weighting. Filled circles with dashed line denote power distribution for 20× after amplitude weighting for FOV centered at (0, 0).

To characterize the properties of our holographic 2P microscope, we acquired 2P images of 1 *μ*m fluorescent beads using the uncaging beam. Fig 3a shows the normalized fluorescence *F* at an uncaging site as a function of the number of foci *N*. It follows the predicted inverse relationship, 1/*N*^2^ [28] (fit: *F* = 1/*N*^1.8±0.02^, *R* = 0.97). 2P excitation is a nonlinear process resulting in a quadratic relation between the fluorescence and excitation intensities [29]. We used this relationship to quantify the uncaging power at an uncaging site from the fluorescence intensity of a bead. To keep the uncaging power per focus constant despite changes in *N*, we fixed the power at a high level and increased *N*. Fig 3b shows how the uncaging power per stimulation spot remains constant as “superfluous” foci were moved outside the holographic FOV. The error in power, *δP*, is calculated as *δP* = 0.5*δF*/*P*. The PSF of 2P excitation with the holographic module measured by imaging 100 nm fluorescent beads shows a full width at half maximum (FWHM) of 0.49 *μ*m (60×) and 0.64 *μ*m (20×) in the lateral direction and 2.7 *μ*m in the axial direction for both lenses. Fig 3c shows the measured axial and transverse PSFs for both the 60× and 20× objective lenses used in our experiments.

We next characterized the holographic FOV—the region within the ROI over which uncaging spots can be positioned in 2D. If an uncaging spot is positioned away from the center of the ROI, the uncaging beam deviates from the optical axis. Beyond a certain deviation, the beam falls off from the relay optics in between the SLM and the scanning mirrors. Fig 3d and 3e show the normalized uncaging power as a function of lateral distance along the *x*-axis for 60× and 20×, respectively. From these plots, we can see that the positioning range of the holographic spots is around 30×30 *μ*m^2^ when using a 60× objective and 70×70 *μ*m^2^ for the 20×. The imaging FOV for the 60× objective is 220×220 *μ*m^2^, while for the 20×, it is 800×800 *μ*m^2^. However, areas within the imaging FOV yet outside the holographic FOV can be easily accessed by steering the uncaging beam using the galvanometer mirrors. In Fig 3d and 3e, when the uncaging beam was steered to other regions (e.g. to (-100,-100) *μ*m and (100,100) *μ*m for 60× and (-300,-300) *μ*m and (300,300) *μ*m for 20×) using the galvanometric mirrors, the holographic FOV was not significantly changed. Within the holographic FOV, spot intensity decreases with lateral displacement as a consequence of a spatially varying diffraction efficiency due to the SLM being pixelated. The intensity decline is in the shape of a sinc-squared function [25]. This non-uniform diffraction efficiency can be compensated for by inversely weighting the amplitude during hologram calculation. Fig 3e shows the amplitude weighted power distribution for the 20× objective with the uncaging beam at the center of the imaging FOV.

To characterize light efficiency in the axial direction, we moved the spot along the *z*-axis and measured the decline in uncaging power. Fig 3f shows the normalized uncaging power as a function of axial distance for both 60× and 20× objectives for different positions of the uncaging beam ((-100,-100), (0,0), (100,100) for 60× and (-300,-300), (0,0), (300,300) for 20×). To counteract this attenuation, the amplitude can be inversely weighted at each position during hologram calculation. Fig 3f shows the amplitude weighted power distribution for the 20× objective with the uncaging beam at the center of the imaging FOV.

The results above show that for the parameters used in our system, photostimulation spots can be generated with a light efficiency of at least 50% (80% with power weighting) over a volume of 70×70×70 *μ*m^3^ when using a 20× objective lens. In our integration experiments (below), the power variation was not significant across the VOI we selected (*x*, *y*, *z* range ∼ 16 *μ*m) and power weighting was not implemented.

**Fig 4:**
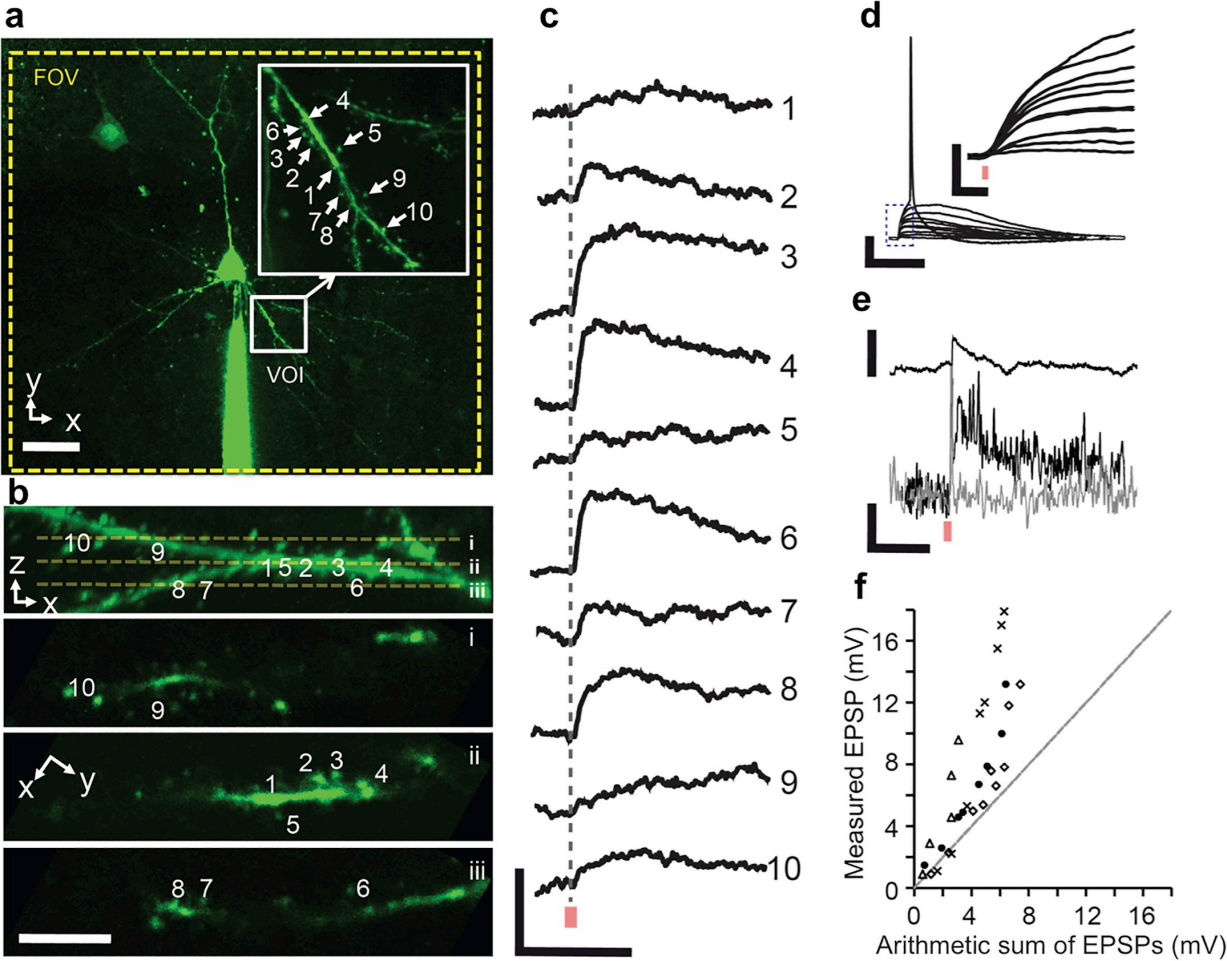
Representative synaptic integration experiment. **a**: Two-photon image of a layer 2/3 pyramidal neuron labeled with OGB-1 taken with a 60× objective. Inset shows a high-magnification flattened multi-stack image of the VOI. Scale bar: 20 *μ*m. **b**: VOI displayed as an *xz*-image with 3 representative image planes (i: *z* = −5 *μ*m; ii: *z* = 0; and iii: *z* = + 5 *μ*m. Scale bar: 5 *μ*m. **c**: Somatic EPSPs of individual uncaging events (red bar indicates time point of 2P glutamate uncaging). Scale bars: 1 mV, 40 ms. **d**: Uncaging responses with increasing number of simultaneous uncaging sites. Scale bars: 20 mV, 50 ms. (Inset) Magnified EPSP rise times for increasing number of uncaging sites. Scale bars: 5 mV, 5 ms. **e**: Representative individual uncaging-evoked EPSP (top) and Ca^2+^ transient (ΔF/F) (bottom) measured at spine 4 (black) and nearby dendrite (gray). Scale bars: 2 mV, 20%, 500 ms. **f**: Input-output plot (*n* = 4) of measured multisite uncaging response against the arithmetic sum of individual uncaging events. Dashed line indicates linear summation, black circles represent data from **a** while all other markers represent data from other experiments.

We then used the holographic projector to perform 2P glutamate uncaging at spines of layer 2/3 pyramidal neurons in brain slices of the somatosensory cortex. Fig 4 shows a representative synaptic integration experiment. A 2P image stack projection of a layer 2/3 pyramidal cell labeled with OGB-1 taken with a 60× objective is shown in Fig 4a. From the image, we selected a specific VOI containing a basal dendrite with a high density of spines. This dendrite featured an arborization with multiple bifurcations that extended to higher-order dendrites with partial overlap along the axial direction (see also S1 and S2 Videos for 3D visualization). We then selected spines and laterally positioned the uncaging sites close to the spines (∼ 0.5 *μ*m). The inset in Fig 4a shows the *z*-projection of the selected VOI displaying the basal dendrite and the locations of the uncaging sites. Fig 4b shows an *xz*-projection of the selected VOI and its 3 representative planes at *z* = −5 *μ*m, *z* = 0, and *z* = + 5 *μ*m. Fig 4c shows the somatic EPSPs of individual uncaging events at spines spanning an axial range of 10 *μ*m. The responses from an increasing number of simultaneous uncaging sites are shown in Fig 4d. An action potential is evoked following simultaneous uncaging at all 10 spines. The inset shows the rising phase of the responses with a higher temporal resolution. Note that only a slight increase of the rise time in the initial phase is observed with increasing number of activated spines.

To demonstrate simultaneous photostimulation and Ca^2+^ imaging, we imaged one plane in the VOI while uncaging with holographic spots. Fig 4e shows a representative individual uncaging EPSP and the corresponding Ca^2+^ transient at the spine (black trace) and dendrite (gray trace). While Ca^2+^ influx is detected at the spine, no change in basal Ca^2+^ concentration is detected at the dendrite. That is, upon uncaging at a single spine, Ca^2+^ invades the spine independent of the dendrite and an individual somatic EPSP is evoked. Fig 4f shows an input-output plot from several experiments (*n* = 4 cells) comparing the uncaging-evoked EPSPs from simultaneous uncaging at multiple sites to the arithmetic sum of individual uncaging EPSPs. Note the supra-linear summation of simultaneous multi-site uncaging events.

**Fig 5:**
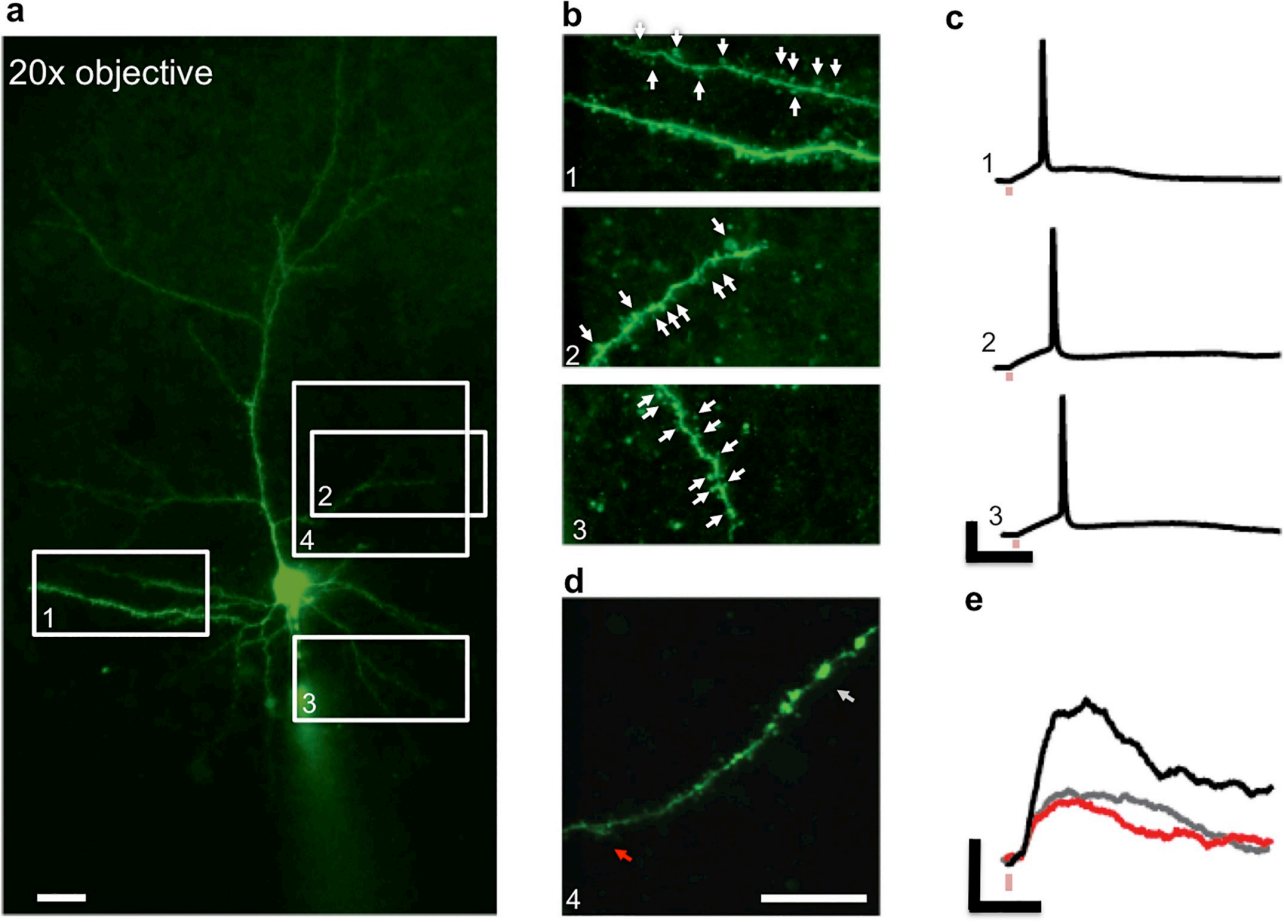
Synaptic integration experiment with a larger VOI. **a**: Two-photon image of a layer 2/3 pyramidal neuron labeled with OGB-1 using a 20× objective lens results in a larger FOV. Scale bar: 20 *μ*m. **b**: Uncaging VOIs (1, 2 and 3) targeting specific dendrites corresponding to regions indicated in **a**. Scale bar: 20 *μ*m. **c**: Somatic action potentials evoked when simultaneously uncaging at all sites (8-10) within corresponding uncaging VOIs in **b**. Scale bars: 20 mV, 50 ms. **d**: Uncaging VOI 4 (region indicated in **a**) showing a dendrite that extends 16 *μ*m along the axial direction. Scale bar: 20 *μ*m. **e**: Evoked EPSPs in VOI 4 when two sites are activated separately (red and grey traces) and simultaneously (black trace). Scale bars: 1 mV, 50 ms.

Next, we show that we can use a lower magnification 20× objective lens to cover a larger VOI. In Fig 5a, we show that we are able to target multiple spines in different regions of the whole dendritic tree of a neuron by steering the uncaging beam using the galvanometer mirrors. Fig 5b shows uncaging VOIs targeting specific dendritic sections as indicated by the ROIs in Fig 5a. By uncaging at 8-10 sites in 2-4 *z* planes in each VOI (1 to 3), we can evoke an action potential as shown in Fig 5c. A representative synaptic integration experiment in the 1^st^ VOI is also shown in S2 Fig. Fig 5d shows the 4^th^ VOI, where we demonstrate a substantial functional *z* range by selecting two spines with an axial distance of 16 *μ*m and activating them separately and simultaneously (Fig 5e).

## Discussion

### Characteristics of the holographic projector module

We have integrated a holographic projector module into a commercial galvanometer-based 2P microscope to introduce arbitrary multi-foci patterns in 3D. This is achieved by encoding a hologram on the excitation laser with a phase-only SLM. To relay the conjugate image of the hologram onto the back focal plane of the objective lens, we needed to use long focal length relay lenses (L5 and L6 in Fig 1b) to match the distance (≈ 500 mm) from the scanning mirrors to the input aperture of our commercial 2P laser scanning system. In the current setup, the positioning range of the holographic spots is limited (70×70 *μ*m^2^ for a 20× objective and 30×30 *μ*m^2^ for 60× objective) due to beam fall-off at the relay lenses. This range can be improved with the use of larger diameter lenses (e.g. ⊘ = 50.8 mm). A microscope with a shorter distance between input aperture and scanning mirrors will require relay lenses with shorter focal length, which can accommodate wide-angle steering of holographic beams and consequently increase the effective holographic FOV.

The current optical design of the holographic projector presents an advantage over our previous configuration [21, 22] where the uncaging beam was not steered by the galvanometer mirrors. In such a configuration, without a relay lens to limit the aperture, the holographic FOV is fundamentally limited by the diffraction efficiency of the SLM. When the SLM image is matched to the size of the back aperture of the objective, the holographic FOV is limited to 2*dx*_*max*_ × 2*dx*_*max*_ where

$$\begin{array}{c} {\text{dx}}_{\text{max}}=\frac{\lambda N_{pix}}{4NA}, \end{array}$$ (2)

where *N*_*pix*_ is the pixel count in the *N_pix_* × *N_pix_* SLM display [30]. Using the parameters of our system, we get a holographic FOV of 220 × 220 *μ*m^2^. Although this FOV is wide, the efficiency of first-order diffraction spots decreases with lateral displacement from the optical axis and follows a sinc-squared-shaped envelope [25, 30]. This non-uniformity can be corrected via inverse amplitude weighting, but this correction in turn reduces the average diffraction efficiency. With the current system, a wide holographic window is conserved in the sense that the holographic FOV can be easily repositioned across the entire imaging FOV with the galvanometer mirrors, allowing for targeted positioning of off-center holographic spots without repositioning the sample. Moreover, the average diffraction efficiency across the smaller holographic FOV is higher. In addition, power loss to far off-axis regions which will have lower average diffraction efficiency can be compensated for by increasing the laser power (or total power of the multiple foci).

The SLM has the unique capability to correct for optical aberrations. We found our holographic system to be primarily affected by oblique astigmatism due to the oblique incidence of the laser on the SLM. However, the amount of aberration was minimal and the improvement brought by correction was insignificant (S3 Fig). Nonetheless, aside from the aberrations introduced by the optics, this functionality is useful for improving the uncaging efficiency when accessing dendrites in deep regions of the brain tissue [31].

### Application of the holographic module: Investigation of synaptic integration in 3D

We have used the system to perform targeted 2P uncaging in spines of L2/3 pyramidal neurons while recording the somatic responses with a patch electrode. Simultaneous uncaging at 8-10 spines across an axial range of up to 16 *μ*m within 0.5-1.5 ms evoked an action potential. We have also shown simultaneous holographic photostimulation and Ca^2+^ imaging in a single plane to study calcium dynamics and monitor a physiological evoked calcium response at the spine. Highly resolved 2P uncaging and calcium imaging at the level of single spines complements the work by Anselmi *et al*. [20], where they introduced remote focusing to perform low resolution 1P uncaging and calcium imaging over a long dendrite that tilts with respect to the optical axis.

The supra-linearity of integration we observed (Fig 4f) in proximal synapses is dependent on the synchrony of inputs [32]. Even in a single plane, the commercial galvanometer-based 2P microscope cannot achieve as many uncaging sites within this time window; despite its fast positioning time (∼ 100 *μ*s), the rate-limiting factor is the dwell time required to release a sufficient amount of glutamate to evoke an EPSP with good signal-to-noise ratio (∼200-500 *μ*s) [10, 33].

The capability of our system to position stimulation sites in 3D offers flexibility in targeting a sufficient number of spines to evoke an action potential or otherwise trigger local regenerative events within dendrites. That is, the required number of spines do not all have to be localized within a single focal plane, an otherwise typical experimental limitation with 2D scanners. While triggering of action potentials has been demonstrated before in 2D for cortical and hippocampal pyramidal neuron dendrites with a high spine density [34, 35], an accessible volume for simultaneous stimulation is particularly desirable for low-density synapse distributions. For example, some spinous cell types feature a much lower spine density than the ∼20 spines/10 *μ*m reported for hippocampal or cortical pyramidal cells [36, 37]. Cortical spinous inhibitory neurons can have as few as 0.3 spines/10 *μ*m depending on the neuron subtype [38]. Likewise, olfactory bulb granule cells feature ∼1-2 spines/10 *μ*m [39]. Moreover, such flexibility can be useful in studies of integration within highly ramified branched structures such as apical tufts of pyramidal neurons or of olfactory bulb mitral and tufted cells. The possibility of moving the VOI without moving the sample could be advantageous for studies of integration across various branch compartments of a cell such as basal versus apical dendrites, in particular for situations where more than one pipette/electrode is positioned in the brain slice such that movement of the sample is limited.

Because the uncaging laser path is integrated into the scanning path, during simultaneous holographic photostimulation and Ca^2+^ imaging, the scanning mirrors are fixed in position for the duration of the uncaging stimulus. The uncaging pulse is 0.5-1.5 ms. This pulse disrupts the Ca^2+^ imaging which is started 700 ms before uncaging but it does not significantly affect the temporal resolution of the imaging as the 10%-90% rise time of the Ca^2+^ indicator we used (OGB-1) is ∼100 ms [40]. Note that even with 10 stimulation sites, the pulse duration remains the same. In contrast, sequential uncaging with the galvanometer mirrors would require up to 5 ms. Thus, there is a shorter disruption to the Ca^2+^ imaging signal with simultaneous multi-site uncaging.

### Comparison with other systems for holographic photostimulation

Holographic photostimulation has been demonstrated previously with 1P uncaging [17–20, 41, 42]. The energy of light used for 1P uncaging (e.g. ultra-violet light) does not require tight focusing and therefore does not suffer in terms of trade-off between FOV and resolution. A 1P uncaging system with extended FOV, however, will have lesser resolution and may not be able to target individual spines. Moreover, 1P uncaging lacks localized photolysis along the axial direction and could uncage unnecessarily at off-target planes. This can be a problem when dendrites are overlapping along the optical axis. Intrinsic localized uncaging in 3D provided by non-linear 2P excitation is preferred in such situations.

To precisely position excitation spots in 3D, multi-site 2P uncaging must also be used in conjunction with high-resolution imaging systems such as confocal or 2P microscopes. Nikolenko *et al*. [15] demonstrated 3D localization and depth focusing of the photostimulation spot using 2P excitation but showed multi-site stimulation only within a single focal plane. We extended this capability to multiple planes [21, 22] by applying a different lens function to the phase hologram of each spot thereby independently focusing each photostimulation spot to a different depth. Both these previous demonstrations of 2P holographic photostimulation employed fully customized microscope systems, while the system described here is modular and can be added into any 2P commercial system with an accessible uncaging light path.

Dal Maschio *et al*. [16] developed a holographic module and integrated it with a commercial scanhead to steer holographically structured illumination patterns for 2P uncaging in 2D. These patterns were used to follow specific contours of cultured neurons similar to the work by Lutz *et al*. [17]. However, a holographically projected shape that covers a larger illumination area results in a poor localization of 2P excitation along the optical axis and is therefore not applicable for stimulating spines along complex dendritic arborizations that overlap along the beam path. While this problem can be solved via temporal focusing [43–45], the use of diffraction-limited 2P stimulation remains a preferred method for multi-site photolysis where each focus stimulates a synaptic input in 3D in a highly localized fashion.

Yang *et al*. [46] also integrated the hologram onto the laser beam of a custom-built 2P scanning microscope for calcium imaging. They used the galvanometer scanning mirrors to sequentially reposition the holographic FOV (140 × 140 *μ*m^2^) 9 times in a tiled manner to achieve an extended FOV of 380 × 380 *μ*m^2^. This is possible for functional calcium imaging since the temporal dynamics of calcium response is in the order of hundreds of milliseconds. However, when used for uncaging over an extended FOV, time-division multiplexing does not achieve simultaneous excitation. In the future, such a large-scale simultaneous excitation may be possible in conjunction with fast SLMs such as those with binary phase shifts using ferroelectric liquid crystals, which can be operated at ∼kHz refresh rates and which could be used to achieve simultaneous multi-site photostimulation in an extended FOV in a biologically relevant time window (a few milliseconds).

Beyond multi-foci uncaging in 3D, our holographic module could also be used for 2P optogenetic activation of neuronal populations with a standard commercial 2P laser scanning microscope [47]. For this application structured illumination (rather than multi-foci patterns) could be used to stimulate and recruit a sufficient number of light-activatable ion channels distributed within the somatic membrane to induce a detectable neuronal response [48].

## Conclusion

We incorporated a holographic projector into a commercial 2P scanning microscope to extend the spatial range of multi-site 2P uncaging. With proper integration of the microscope’s built-in 3D rendering, zoom and arbitrary ROI function, simultaneous 2P uncaging at multiple sites can be performed at highly resolved sections targeting spines of dendritic trees branching in 3D, with a number of inputs sufficient for eliciting action potentials from rest. Moreover, the combination allowed the positioning of the holographic VOI arbitrarily across the entire imaging FOV without repositioning the sample. Attaching a holographic projector to a commercial 2P microscope offers 3D multi-site photostimulation with the advantages of versatility and straightforward implementation over custom 2P holographic systems and will be an important tool in the study of synaptic integration.

## Supporting information

S1 Fig Calibration and aberration correction of excitation spots. Custom SLM software GUI for **a**: aberration correction and **b**: calibration of uncaging spots.
(JPG)

S2 Fig Synaptic integration experiment with a 20× objective lens. **a**: Two-photon image of a layer 2/3 pyramidal cell labeled with OGB-1. Scale bar 20 *μ*m. **b**: The VOI displayed as an *xz*-image with 3 representative image planes (i: *z* = −2 *μ*m; ii: *z* = 0; and iii: *z* = + 4 *μ*m. Scale bar: 20 *μ*m. **c**: Somatic EPSPs of individual uncaging events (red bar indicates time point of 2P glutamate uncaging). Scale bars: 1 mV, 40 ms. **d**: Uncaging responses with increasing number of simultaneous uncaging sites. Scale bars: 20mV, 50 ms. (Inset) Magnified EPSP rise times for increasing number of uncaging sites. Scale bars: 5 mV, 5 ms. **e**: Representative individual uncaging-evoked EPSP at spine 4 and corresponding Ca^2+^ transient (ΔF/F) in spine (black) and nearby dendrite (gray). Scale bars: 2 mV, 20%, 500 ms.
(JPG)

S3 Fig Aberration correction. Effect of correction for oblique astigmatism on the 2P fluorescence intensity (8-bit gray level) of a fluorescent bead for different numbers of foci. (Inset) 2P images of a fluorescent bead with no correction and with correction for oblique astigmatism.
(JPG)

S1 Video 3D rendering of VOI. Video showing rotation of the VOI used in the experiment in Fig 4 along the y-axis. Shown is the 3D extension of the segment of dendrite targeted for multi-site uncaging.
(AVI)

S2 Video 3D rendering showing various planes of VOI. Video showing different axial planes of the segment of dendrite targeted for multi-site uncaging in the experiment in Fig 4.
(AVI)

## References

[1] Svoboda K, Yasuda R. Principles of two-photon excitation microscopy and its applications to neuroscience. Neuron. 2006 6;50(6):823–839. 10.1016/j.neuron.2006.05.019 16772166

[2] Denk W, Delaney KR, Gelperin A, Kleinfeld D, Strowbridge BW, Tank DW, et al Anatomical and functional imaging of neurons using 2-photon laser scanning microscopy. J Neurosci Methods. 1994 10;54(2):151–162. 10.1016/0165-0270(94)90189-9 7869748

[3] Grienberger C, Konnerth A. Imaging calcium in neurons. Neuron. 2012 3;73(5):862–885. 10.1016/j.neuron.2012.02.011 22405199

[4] Fisher JA, Salzberg BM. Two-photon excitation of fluorescent voltage-sensitive dyes: monitoring membrane potential in the infrared In: Canepari M, Zecevic D, Bernus O, editors. Membrane potential imaging in the nervous system and heart. Cham: Springer International Publishing; 2015 pp. 427–453. 10.1007/978-3-319-17641-3_17 26238063

[5] Kramer RH, Fortin DL, Trauner D. New photochemical tools for controlling neuronal activity. Curr Opin Neurobiol. 2009 10;19(5):544–552. 10.1016/j.conb.2009.09.004 19828309 PMC2788492

[6] Jerome J, Heck DH. The age of enlightenment: evolving opportunities in brain research through optical manipulation of neuronal activity. Front Sys Neurosci. 2011 12;5:95 10.3389/fnsys.2011.00095 PMC3257845 22275886

[7] Denk W, Strickler JH, Webb WW. Two-photon laser scanning fluorescence microscopy. Science. 1990 4;248(4951):73–76. 10.1126/science.2321027 2321027

[8] Lillis KP, Eng A, White JA, Mertz J. Two-photon imaging of spatially extended neuronal network dynamics with high temporal resolution. J Neurosci Methods. 2008 7;172(2):73–76. 10.1016/j.jneumeth.2008.04.024 PMC2582024 18539336

[9] Fan GY, Fujisaki H, Miyawaki A, Tsay RK, Tsien RY, Ellisman MH. Video-rate scanning two-photon excitation fluorescence microscopy and ratio imaging with cameleons. Biophys J. 1999 5;76(5):2412–2420. 10.1016/S0006-3495(99)77396-0 10233058 PMC1300213

[10] Losavio BE, Iyer V, Saggau P. Two-photon microscope for multisite microphotolysis of caged neurotransmitters in acute brain slices. J Biomed Opt. 2009 12;14(6):064033 10.1117/1.3275468 20059271 PMC2809696

[11] Reddy GD, Kelleher K, Fink R, Saggau P. Three-dimensional random access multiphoton microscopy for functional imaging of neuronal activity. Nat Neurosci. 2008 6;11(6):713–720. 10.1038/nn.2116 18432198 PMC2747788

[12] Kirkby PA, Nadella KMNS, Silver RA. A compact acousto-optic lens for 2D and 3D femtosecond based 2-photon microscopy. Opt Express. 2010 6;18(13):13721–13745. 10.1364/OE.18.013720 20588506 PMC2948528

[13] Nadella KMNS, Ros̆ H, Baragli C, Griffiths VA, Konstantinou G, Koimtzis T, et al Random-access scanning microscopy for 3D imaging in awake behaving animals. Nature Methods. 2016 10;13:1001–1004. 10.1038/nmeth.4033 27749836 PMC5769813

[14] Nikolenko V, Poskanzer KE, Yuste R. Two-photon photostimulation and imaging of neural circuits. Nature Methods. 2007 10;4:943:950 10.1038/nmeth1105 17965719

[15] Nikolenko V, Watson BO, Araya R, Woodruff A, Peterka DS, Yuste R. SLM microscopy: scanless two-photon imaging and photostimulation with spatial light modulators. Front Neural Circuits. 2008 12;2:5 10.3389/neuro.04.005.2008 19129923 PMC2614319

[16] Dal Maschio M, Difato F, Beltramo R, Blau A, Benfenati F, Fellin T. Simultaneous two-photon imaging and photostimulation with structured light illumination. Opt Express. 2010 8;18(18):18720–18731. 10.1364/OE.18.018720 20940765

[17] Lutz C, Otis TS, DeSars V, Charpak S, DiGregorio DA, Emiliani V. Holographic photolysis of caged neurotransmitters. Nat. Methods. 2008 9; 5(9):821–7. 10.1038/nmeth.1241 19160517 PMC2711023

[18] Yang S, Papagiakoumou E, Guillon M, de Sars V, Tang CM, Emiliani V. Three-dimensional holographic photostimulation of the dendritic arbor. J Neural Eng. 2011 5;8:046002 10.1088/1741-2560/8/4/046002 21623008

[19] Yang S, Emiliani V, Tang CM, The kinetics of multibranch integration on the dendritic arbor of CA1 pyramidal neurons. Front. Cell. Neurosci. 2014 5; 8:127 10.3389/fncel.2014.00127 24860429 PMC4026731

[20] Anselmi F, Ventalon C, Bègue A, Ogden D, Emiliani V. Three-dimensional imaging and photostimulation by remote-focusing and holograpihc light patterning. Proc Natl Acad Sci. 2011 12;108(49):19504–19509. 10.1073/pnas.1109111108 22074779 PMC3241782

[21] Go MA, Stricker C, Redman S, Bachor HA, Daria VR. Simultaneous multi-site two-photon photostimulation in three dimensions. J Biophotonics. 2012 10;5(10):745–753. 10.1002/jbio.201100101 22345073

[22] Go MA, To MS, Stricker C, Redman S, Bachor HA, Stuart GJ, et al Four-dimensional multi-site photolysis of caged neurotransmitters. Front Cell Neurosci. 2013 12;7:231 10.3389/fncel.2013.00231 24348330 PMC3845713

[23] Liesener J, Reichester M, Haist T, Tiziani HJ. Multi-functional optical tweezers using computer generated holograms. Opt Commun. 2000 11;185:77–82. 10.1016/S0030-4018(00)00990-1

[24] Curtis J, Koss BA, Grier D. Dynamic holographic optical tweezers. Opt Commun. 2000 6;207:169–175. 10.1016/S0030-4018(02)01524-9

[25] Golan L, Reutsky I, Farah N, Shoham S. Design and characteristics of holographic neural photo-stimulation systems. J Neural Eng. 2009 12;6(6):066004 10.1088/1741-2560/6/6/066004 19837999

[26] Born M, Wolf E. Principles of optics: electromagnetic theory of propagation, interference, and diffraction of light. Pergamon Press; 1989.

[27] Bywalez WG, Patirniche D, Rupprecht V, Stemmler M, Herz AVM, Pálfi D, et al Local postsynaptic voltage-gated sodium channel activation in dendritic spines of olfactory bulb granule cells. Neuron. 2015 1;85(3):590–601. 10.1016/j.neuron.2014.12.051 25619656

[28] Daria VR, Stricker C, Bowman R, Redman S, Bachor HA. Arbitrary multisite two-photon excitation in four dimensions. Appl Phys Lett. 2009 9;95(9):093701 10.1063/1.3216581

[29] Zipfel WR, Williams RM, Webb WW. Nonlinear magic: multiphoton microscopy in the biosciences. Nature Biotechnol. 2003 10;21(11):1369–1377. 10.1038/nbt899 14595365

[30] van der Horst A, Forde NR. Calibration of dynamic holographic optical tweezers for force measurements on biomaterials. Opt Express. 2008 12;16(25):20987 10.1364/OE.16.020987 19065239

[31] Choy JMC, Sané S, Lee WM, Stricker C, Bachor HA, Daria VR. Improving focal photostimulation of cortical neurons with pre-derived wavefront correction. Front Cell Neurosci. 2017 5;207:11–105. 10.3389/fncel.2017.00105 PMC5410561 28507508

[32] Branco T, Häusser M. Synaptic integration gradients in single cortical pyramidal cell dendrites. Neuron. 2011 3;69(5):885–892. 10.1016/j.neuron.2011.02.006 21382549 PMC6420135

[33] Losonczy A, Makara JK, Magee JC. Compartmentalized dendritic plasticity and input feature storage in neurons. Nature. 2008 3;452:436–441. 10.1038/nature06725 18368112

[34] Branco T, Clark BA, Häusser M. Dendritic discrimination of temporal input sequences in cortical neurons. Science. 2010 9 24;329(5999):1671–5. 10.1126/science.1189664 20705816 PMC6354899

[35] Makara JK, Magee JC. Variable dendritic integration in hippocampal CA3 pyramidal neurons. Neuron. 2013 12 18; 80(6): 1438–1450. 10.1016/j.neuron.2013.10.033 24360546 PMC3878388

[36] Harris KM, Stevens JK. Dendritic Spines of CA1 Pyramidal Cells in the Rat Hippocampus: Serial Electron Microscopy with Reference to Their Biophysical Characteristics. J Neuroscience. 1989 8;9(8): 2982–2997. 10.1523/JNEUROSCI.09-08-02982.1989 PMC6569708 2769375

[37] Ballesteros-Yáñez I, Benavides-Piccione R, Elston GN, Yuste R, DeFelipe J. Density and morphology of dendritic spines in mouse neocortex. Neuroscience. 2006 2;138(2):403–9. 10.1016/j.neuroscience.2005.11.038 16457955

[38] Kawaguchi Y, Karube F, Kubota Y. Dendritic branch typing and spine expression patterns in cortical nonpyramidal cells. Cereb Cortex. 2006 5;16(5):696–711. 10.1093/cercor/bhj015 16107588

[39] Saghatelyan A, Roux P, Migliore M, Rochefort C, Desmaisons D, Charneau P, et al Activity-dependent adjustments of the inhibitory network in the olfactory bulb following early postnatal deprivation. Neuron. 2005 4;46(1):103–16. 10.1016/j.neuron.2005.02.016 15820697

[40] Tada M, Takeuchi A, Hashizume M, Kitamura K, Kano M. A highly sensitive fluorescent indicator dye for calcium imaging of neural activity *in vitro* and *in vivo*. Eur J Neurosci. 2014 6;39(11):1720–1728. 10.1111/ejn.12476 24405482 PMC4232931

[41] Yang S, Yang S, Moreira T, Hoffman G, Carlson GC, Bender KJ, et al Interlamellar CA1 network in the hippocampus. Proc Natl Acad Sci. 2014 9;111(35):12919–129249. 10.1073/pnas.1405468111 25139992 PMC4156755

[42] Tanese D, Weng JY, Zampini V, De Sars V, Canepari M, Rozsa B, et al Imaging membrane potential changes from dendritic spines using computer-generated holography. Neurophotonics. 2017 7;4(3):031211 10.1117/1.NPh.4.3.031211 28523281 PMC5428833

[43] Oron D, Tal E, Silberberg Y. Scanningless depth resolved microscopy. Opt Express. 2005 3;13(5):1468–1476. 10.1364/OPEX.13.001468 19495022

[44] Hernandez O, Papagiakoumou E, Tanese D, Fidelin K, Wyart C, Emiliani V. Three-dimensional spatiotemporal focusing of holographic patterns. Nat Commun. 2016 6;7:11928 10.1038/ncomms11928 27306044 PMC4912686

[45] Pégard NC, Mardinly AR, Oldenburg IA, Sridharan S, Waller L, Adesnik H. Three-dimensional scanless holographic optogenetics with temporal focusing (3D-SHOT). Nat Commun. 2017 10;8:1228 10.1038/s41467-017-01031-3 29089483 PMC5663714

[46] Yang SJ, Allen WE, Kauvar I, Andalman AS, Young NP, Kim CK, et al Extended field-of-view and increased-signal 3D holographic illumination with time-division multiplexing. Opt Express. 2015 12;23(25):32573–32581. 10.1364/OE.23.032573 26699047 PMC4775739

[47] Yang W, Carrillo-Reid L, Bando Y, Peterka DS, Yuste R. Simultaneous two-photon imaging and two-photon optogenetics of cortical circuits in three dimensions. eLife. 2018 2;7:e32671 10.7554/eLife.32671 29412138 PMC5832414

[48] Rickgauer JP, Tank DW. Two-photon excitation of channelrhodopsin-2 at saturation. Proc Natl Acad Sci U S A. 2009 9 1;106(35):15025–30. 10.1073/pnas.0907084106 19706471 PMC2736443

