# Supplementary figures and images for: A compact holographic projector module for high-resolution 3D multi-site two-photon photostimulation
Source: PLoS One. 2019 Jan 28;14(1):e0210564. doi: 10.1371/journal.pone.0210564 (PMC6349413; doi:10.1371/journal.pone.0210564)

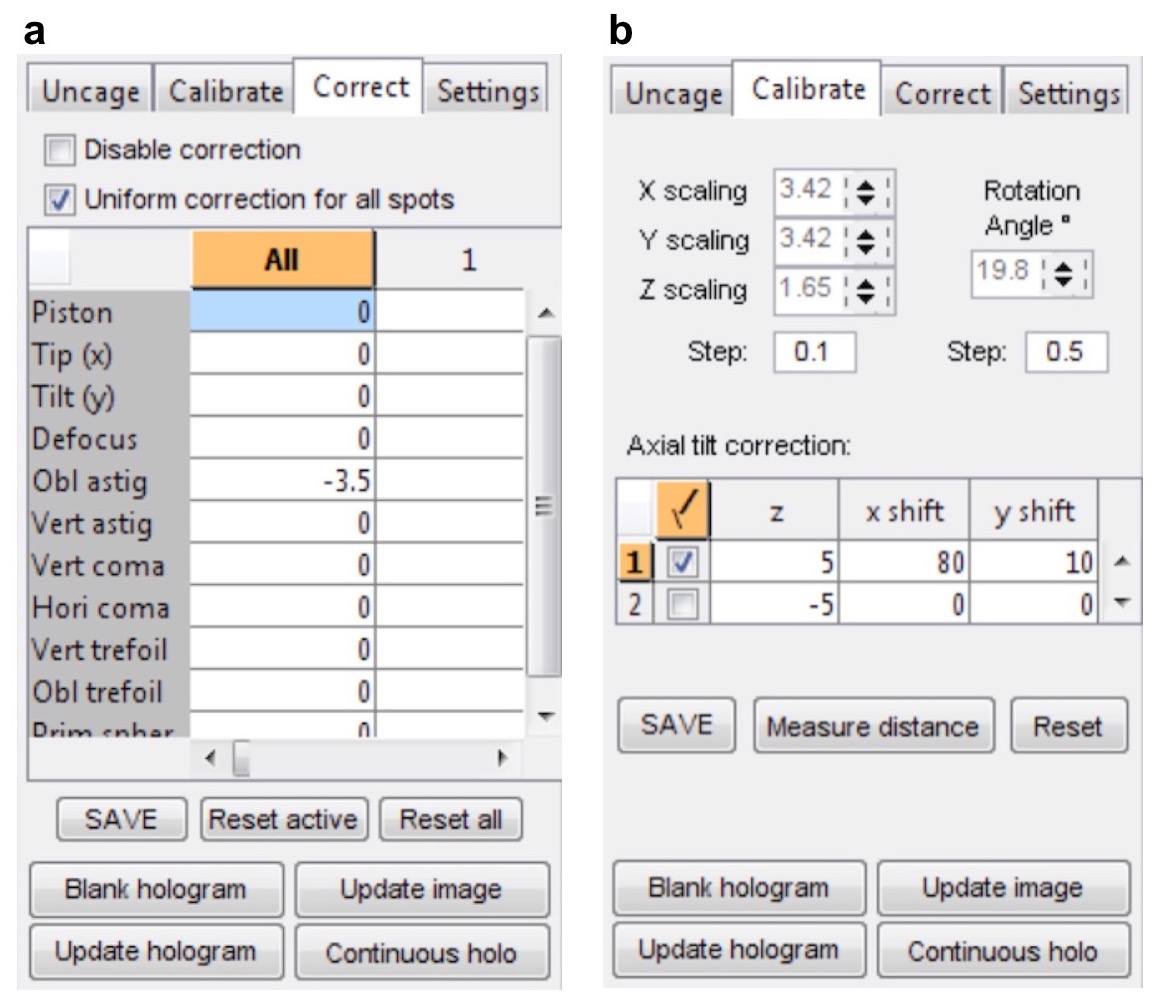

Supplement: S1 Fig — Custom SLM software GUI for a: aberration correction and b: calibration of uncaging spots. (JPG) [file pone.0210564.s001.jpg]

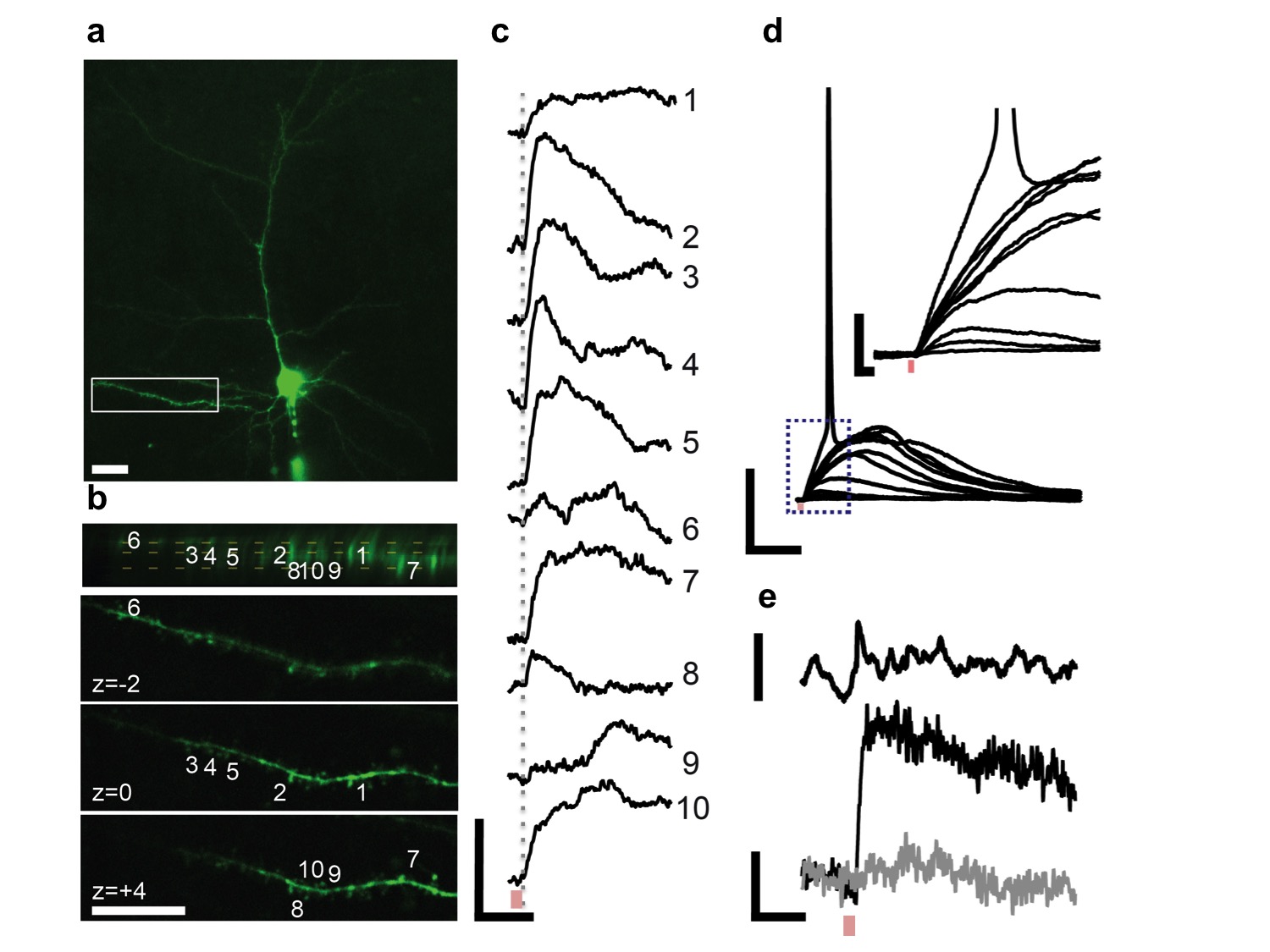

Supplement: S2 Fig — a: Two-photon image of a layer 2/3 pyramidal cell labeled with OGB-1. Scale bar 20 μm. b: The VOI displayed as an xz-image with 3 representative image planes (i: z = −2 μm; ii: z = 0; and iii: z = + 4 μm. Scale bar: 20 μm. c: Somatic EPSPs of individual uncaging events (red bar indicates time point of 2P glutamate uncaging). Scale bars: 1 mV, 40 ms. d: Uncaging responses with increasing number of simultaneous uncaging sites. Scale bars: 20mV, 50 ms. (Inset) Magnified EPSP rise times for increasing number of uncaging sites. Scale bars: 5 mV, 5 ms. e: Representative individual uncaging-evoked EPSP at spine 4 and corresponding Ca2+ transient (ΔF/F) in spine (black) and nearby dendrite (gray). Scale bars: 2 mV, 20%, 500 ms. (JPG) [file pone.0210564.s002.jpg]

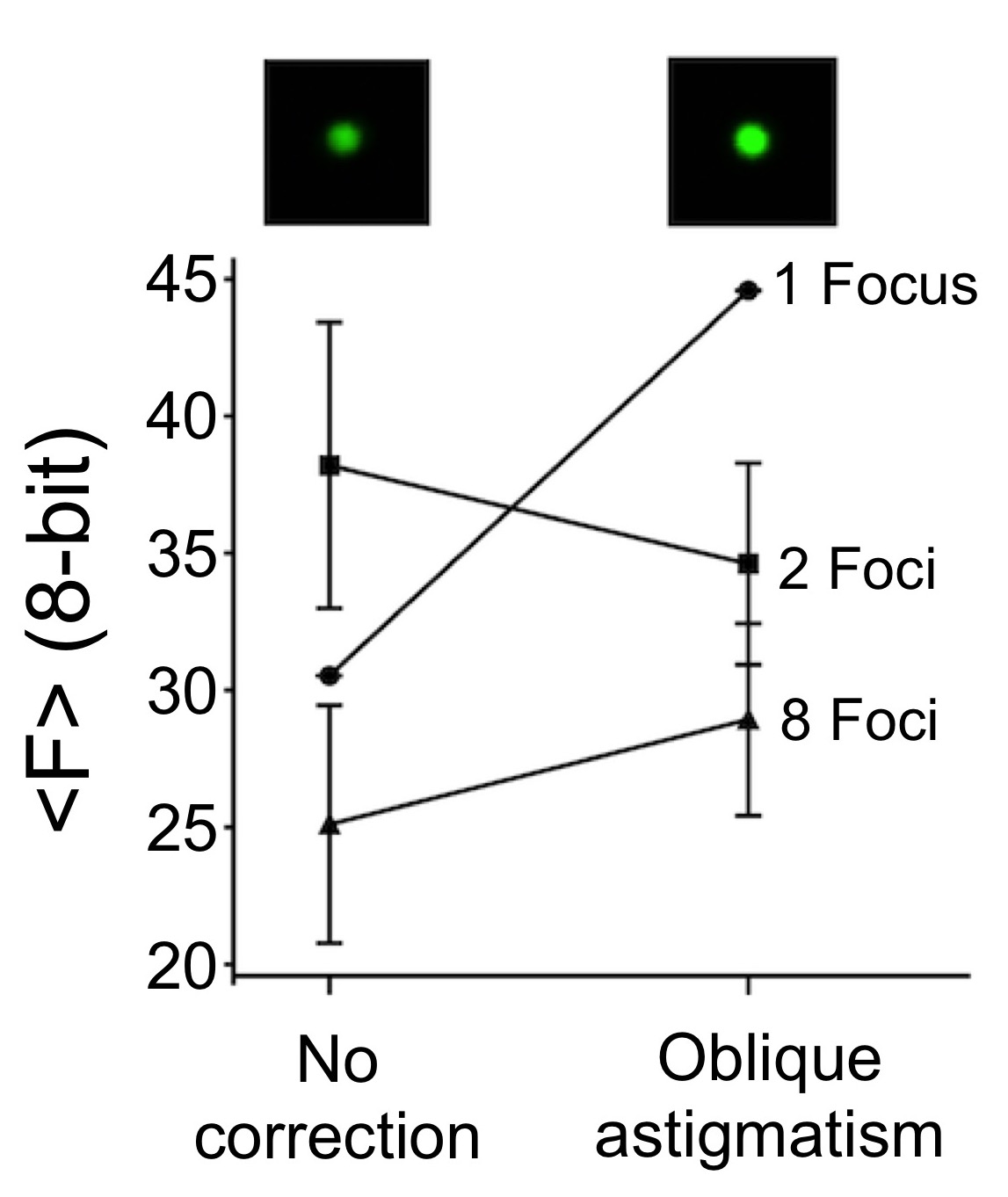

Supplement: S3 Fig — Effect of correction for oblique astigmatism on the 2P fluorescence intensity (8-bit gray level) of a fluorescent bead for different numbers of foci. (Inset) 2P images of a fluorescent bead with no correction and with correction for oblique astigmatism. (JPG) [file pone.0210564.s003.jpg]
